# Benzoxazole-Based Metal Complexes to Reverse Multidrug Resistance in Bacteria

**DOI:** 10.3390/antibiotics9100649

**Published:** 2020-09-28

**Authors:** Annamária Kincses, Stefánia Szabó, Bálint Rácz, Nikoletta Szemerédi, Genki Watanabe, Ryosuke Saijo, Hiroshi Sekiya, Eiji Tamai, Joseph Molnár, Masami Kawase, Gabriella Spengler

**Affiliations:** 1 Department of Medical Microbiology and Immunobiology, Faculty of Medicine, University of Szeged, Dóm tér 10, H-6720 Szeged, Hungary; stefiszabo@gmail.com (S.S.); balintracz95@gmail.com (B.R.); szemeredi.nikoletta@med.u-szeged.hu (N.S.); molnar.jozsef@med.u-szeged.hu (J.M.); spengler.gabriella@med.u-szeged.hu (G.S.); 2 College of Pharmaceutical Sciences, Matsuyama University, 4-2 Bunkyo-cho, Matsuyama 790-8578, Japan; gykks333w@icloud.com (G.W.); rsaijo@g.matsuyama-u.ac.jp (R.S.); kawase@g.matsuyama-u.ac.jp (M.K.); 3 Department of Infectious Diseases, College of Pharmaceutical Sciences, Matsuyama University, 4-2 Bunkyo-cho, Matsuyama 790-8578, Japan; hsekiya@g.matsuyama-u.ac.jp (H.S.); etamai@g.matsuyama-u.ac.jp (E.T.)

**Keywords:** *E. coli*, *Staphylococcus aureus*, multidrug resistance, benzoxazole skeleton, metal complexes, efflux pump, biofilm

## Abstract

Bacteria often show resistance against antibiotics due to various mechanisms such as the expression of efflux pumps, biofilm formation, or bacterial quorum sensing (QS) controls. For successful therapy, the discovery of alternative agents is crucial. The objective of this study was to evaluate the efflux pump, anti-biofilm, and QS inhibiting, as well as antibacterial effects of 2-trifluoroacetonylbenzoxazole ligands (**1–3**) and their metal complexes (**4–12**) in bacteria. The ligand **2** and its Zn(II) complex **5**, and furthermore the Cu(II) complex **7** of ligand **1**, exerted remarkable antibacterial activity on the *Staphylococcus aureus* 272123 (MRSA) strain. In the minimum inhibitory concentration (MIC) reduction assay the ligand **3**, the Zn(II) complex **5** of ligand **2**, and the Cu(II), Ni(II), Mg(II), Fe(III) complexes (**7**, **8**, **9**, **12**) of ligand **1** enhanced the antibacterial activity of ciprofloxacin in MRSA. An increased ethidium bromide accumulation was detected for ligand **3** in MRSA while the Fe(III) complex **12** of ligand **1** decreased the biofilm formation of the reference *S. aureus* ATCC 25923 strain. The Zn(II) and Ag(II) complexes (**3** and **4**) of ligand **1** and ligand **3** inhibited the QS. Based on our results, the ligands and their metal complexes could be potential alternative drugs in the treatment of infectious diseases.

## 1. Introduction

Antimicrobial resistance is one of the greatest concerns of public health worldwide since drug-resistant bacterial infections have increased risk of worse clinical outcomes and death. The infection caused by resistant bacteria results in lengthier stays in hospital and may need more intensive care or more expensive drugs [1]. Antibacterial resistance can come from various mechanisms, including enzymatic degradation of antibiotics, alteration of antibiotic targets, blocking of drug entry, and increasing expression of efflux pumps (EPs) [2].

EPs play a significant role in bacteria; six different families of EP systems enable bacteria to extrude various potentially toxic substances such as antibiotics, heavy metals, organic pollutants, and plant-produced compounds. Among these families we can differentiate: ATP-binding cassette (ABC) transporters, multidrug and toxic compound extrusion (MATE) transporters, small multidrug resistance (SMR) transporters, major facilitator superfamily (MFS) transporters, resistance nodulation cell division (RND) transporters, and proteobacterial antimicrobial compound efflux (PACE) transporters [3]. MFS and MATE transporters are expressed mainly by Gram-positive bacteria, while RND transporters are found almost exclusively in Gram-negative bacteria [4]. The most investigated RND efflux system is AcrAB-TolC, which constitutes the AcrB RND EP, TolC outer membrane protein, and AcrA serves as periplasmic adaptor protein [5].

The formation of biofilms gives rise to many clinical problems and plays a role in antibacterial resistance. Biofilm is a dynamic structure built by multicellular bacterial community and its extracellular polymeric matrix. This environment not only provides bacteria with protection against drugs, host immune attacks, and environmental stresses, but also forms a platform of metabolic exchange and cellular communication [6].

Quorum sensing (QS) is a cell–cell communication system enabling bacteria to regulate gene expression based on cell population density [7]. Though QS bacteria can alter their phenotypes and behaviors, they may be able to form biofilms and express various virulence factors (such as EPs) resulting in an enhanced drug resistance [8].

Metal complexes are already widely used in clinical settings and many antibiotics containing metal complexes have better pharmaceutical characteristics [9,10,11]. Benzoxazole skeleton-containing molecules are also in the scope of research due to their antimicrobial and antiproliferative properties [12,13,14]. The importance of benzoxazole core and 2-trifluoroacetonyl moiety of 2-trifluoroacetonylbenzoxazole **1** in order to express their antimicrobial activities has been previously highlighted [15,16,17]. In recent work on 2-trifluoroacetonylbenzoxazole ligand **1** for metal complexes, Zn(II) complex **4** of ligand **1** formed a stable six-membered ring in a bidentate mode to consist of **1** with Zn(II) ion in 2:1 ratio with good antibacterial activities against Gram-positive pathogens [18] and potent multidrug-resistance reversing activities in cancer cells [19]. In this article, we report the multidrug resistance-modulating activities of 2-trifluoroacetonylbenzoxazole ligands (**1–3**) and their metal complexes (**4–12**) in bacteria related to the inhibition of QS and biofilm formation.

## 2. Results

### 2.1. Antibacterial Activity

The antibacterial activity of 2-trifluoroacetonylbenzoxazole ligands and their metal complexes was tested on two Gram-positive (*Staphylococcus aureus* ATCC 25923 and *S. aureus* 272123 (MRSA), two Gram-negative (wild-type *Escherichia coli* AG100 expressing the AcrAB TolC EP at its basal level and its AcrAB-TolC deleted mutant *E. coli* AG100 A strain), and QS (*Chromobacterium violaceum* 026 and *Enterobacter cloacae* 31298) bacterial strains. Most compounds were effective against *S. aureus* and *E. coli* strains and the most potent effects were detected on the MRSA strain (Table 1).

The ligand **2** containing a CF_2_Cl moiety and its Zn(II) complex **5** furthermore Cu(II) complex **7** of ligand **1** had the strongest antibacterial activity (minimum inhibitory concentration (MIC): 3.125 μM) on *S. aureus* MRSA. The MIC value of ligand **3** containing a CF_2_H moiety was 50 μM, while its Zn(II) complex **6** exhibited an MIC of 12.5 μM on the MRSA strain. Furthermore, ligand **1** showed an MIC of 12.5 μM; however, the ligand in Cu(II) complex **7** increased its activity, showing an MIC of 3.125 μM. In addition, the complexes of ligand **1** with Zn (**4**), Ni (**8**), and Mg (**9**) were also more effective than ligand **1** alone against MRSA. None of the compounds had any antibacterial effect on the *C. violaceum* and *E. cloacae* strains. The MIC of solvent dimethyl sulfoxide (DMSO) was >1%, and the concentration of DMSO applied in the assay had no antibacterial effect.

### 2.2. Resistance Modulation Assay

The resistance modulation effects of compounds with ciprofloxacin (CIP) and tetracycline (TET) antibiotics were evaluated by the MIC reduction method on the Gram-negative *E. coli* AG100, AG100 A, and the Gram-positive *S. aureus* ATCC and MRSA strains. In the absence of the compounds, the MIC value of CIP was 12.5 µM on MRSA and 0.016 µM on *E. coli* AG100. Ligand **3**, the Zn(II) complex **5** of ligand **2**, and the complex of ligand **1** with Cu (**7**), Ni (**8**), Mg (**9**), and Fe (**12**) showed synergism with CIP: these compounds applied at a quarter of the MIC reduced the MIC value of CIP by 2-fold (6.25 μM) on MRSA (Table 2).

The Ni(II) and Mg(II) complexes (**8** and **9**) of ligand **1** were able to decrease the MIC of CIP, resulting in a 2-fold reduction on *E. coli* AG100 (Table 2). None of the compounds could modulate the MIC of TET on the strains investigated; furthermore, the compounds were not able to improve the activity of CIP on the reference *S. aureus* ATCC and *E. coli* AG100 A strains (data not shown).

### 2.3. Efflux Pump Inhibiting Activity

Since multidrug resistance EPs are very important factors contributing to multidrug resistance (MDR) in both Gram-positive and Gram-negative bacteria, the inhibition of these pumps could reverse the resistant phenotype and restore the activity of antibiotics. The effect of compounds on ethidium bromide (EB) accumulation was determined by the automated EB method. This assay was used to determine how much of the fluorescent dye and pump substrate EB was accumulated by the bacterial cells in the presence of the tested compounds. The real-time accumulation of EB was monitored for 30 min in both *S. aureus* and *E. coli* strains. Ligand **3** was a potent EP inhibitor (relative fluorescence index or RFI: 0.13) at 12.5 µM compared to the positive control verapamil (VER) (RFI: 0.14) at 25 µM in MRSA; on the other hand, the other ligands and complexes had no effect on the intracellular EB accumulation in both *S. aureus* and *E. coli* AG100 A strains (Table 3).

However, the Zn(II) complex **5** of the ligand **2** (RFI: 0.37) and the Ag(I) complex **11** of ligand **1** (RFI: 0.37) compared with the positive control chlorpromazine (CPZ) (RFI: 0.38), exhibited remarkable inhibition on the AcrAB-TolC in *E. coli* AG100. The other compounds had no activity on the intracellular EB accumulation by *E. coli* AG100 (Table 3).

### 2.4. Anti-Biofilm Activity

**Figure 1:**
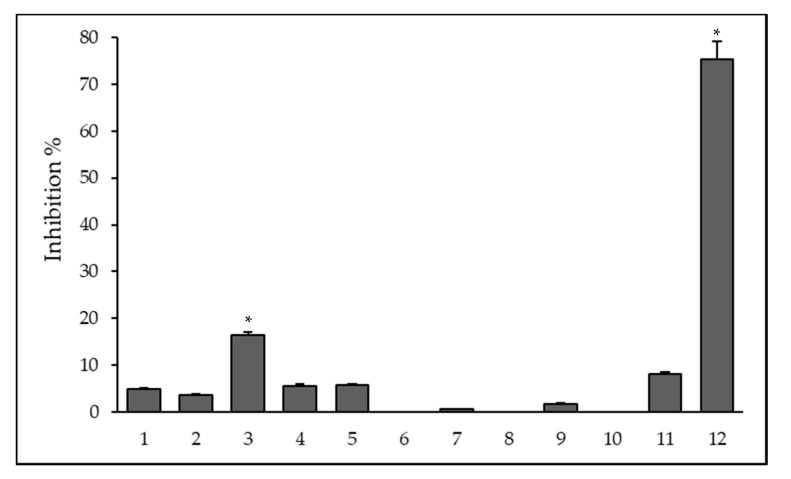
Anti-biofilm effect of ligands and their metal complexes on *Staphylococcus aureus* ATCC 25923 at ¼ MIC. The level of significance was * *p* < 0.001.

The effect of ligands and their metal complexes on biofilm formation was evaluated on the reference *S. aureus* ATCC 25923 strain. The biofilm inhibition (%) was calculated based on the mean of absorbance units. The decrease of biofilm formation was calculated in comparison to the untreated biofilm mass and the percentage of inhibition was calculated based on these data, the level of significance was *p* < 0.001. As shown in Figure 1, the ligand **3** and the Fe(III) complex **12** of ligand **1** exhibited biofilm inhibition of 16.4 and 75%, respectively.

### 2.5. Inhibition of Quorum Sensing

As shown in Table 4, the most remarkable inhibition of quorum sensing was exerted by ligand **3** (inhibition zone: 56 mm) and the Ag(I) complex **11** of ligand **1** followed by the Zn(II) complex **4** of ligand **1** (inhibition zone: 47 mm). In addition, the Zn(II) complex **5** of ligand **2** exerted a similar anti-QS effect to the positive control promethazine (PMZ), leading to the decrease of violacein production. The other ligand alone and the other metal complexes did not possess any effects to inhibit the bacterial communication (inhibition zone: 0 mm; data not shown).

### 2.6. Cytotoxic Effect on Human Cell Line

The toxic effect of ligands and their metal complexes were tested on the MRC-5 human embryonal lung fibroblast cell line by cytotoxicity assay. Zn(II) and Ni(II) complexes (**4** and **8**) of ligand **1**, Zn(II) complex **5** of ligand **2**, and the Zn(II) complex **6** of ligand **3** had a strong cytotoxic effect on human cells (IC_50_ between 4.72 and 24.57 µM). Ligand **3** alone presented moderate inhibitory activity; the IC_50_ value was 44.45 µM. The complex of ligand **1** with Cu (**7**), Pd (**10**), and Ag (**11**) showed weak cytotoxic activity on MRC-5. Fortunately, ligand **1** alone and its complexes with Mg (**9**), Fe (**12**), and furthermore ligand **2**, had no toxic effect on normal cells: Their IC_50_ values were >100 µM (Table 5).

## 3. Discussion

Metal complexes could provide a beneficial tool for pharmaceuticals and there have been several compounds with anticancer, antimalarial, and anti-neurodegenerative activities in clinical trials. However, metal complexes as potential antibacterial agents gained less attention until now, despite the fact that they possess remarkable antimicrobial effects [20]. Based on our results, the antibacterial effect of 2-fluoroacetonylbenzoxazole ligands could be increased by metal-based coordination complexes of these ligands. The metal complexes could be applied to reduce the virulence of bacteria (inhibition of QS and biofilm formation) and restore the sensitivity of multidrug resistant bacteria by modulating the overexpressed EP systems, and they could be administered together with antibiotics to improve the efficacy of antibacterial therapy.

As shown previously, transition metal complexes can demonstrate better antimicrobial activity than the ligands themselves. 2-substituted benzoxazoles are important scaffolds because they possess antimicrobial, antiviral, antifungal, and anticancer effects as well [18].

Based on the results, it has been confirmed that the metal complexes exerted more pronounced antibacterial activity than the ligands: This tendency was observed in the case of the complexes of the 2-trifluoroacetonylbenzoxazole ligand **1** with Zn(II), Cu(II), Ni(II), Mg(II), and, furthermore, in the case of the complex of the ligand **3** with Zn(II) on the Gram-positive *S. aureus* MRSA and reference ATCC strains. However, ligand **2** and with Zn(II), furthermore, ligand **1** with Cu(II), had a more potent antibacterial effect (MIC: 3.125 µM) than the reference standard TET (MIC: 6.25 µM) or CIP (MIC: 12.5 µM) on the *S. aureus* MRSA strain. Ligand **1** with Zn(II), Ni(II), and Mg(II) were more effective (MIC: 6.25 µM) compared to CIP against *S. aureus* MRSA. The MIC of TET was 50 µM on *E. coli* AG100, but stronger growth inhibition was observed for ligand **1** with Zn(II) and Ag(II); MIC was 25 µM.

In three ligands **1**–**3**, the order of antibacterial potency was **2** > **1**> **3**. Interestingly, ligand **3** with the lowest antibacterial activity could potentiate the activity of CIP on MRSA strain. The other ligands **1** and **2** could show the same activity in the form of metal complexes: the Zn(II) complex **5** of ligand **2** and the complexes of ligand **1** with Cu (**7**), Ni (**8**), Mg (**9**), and Fe (**12**). In addition, complexes **8** and **9** were effective in combination with CIP on *E. coli* AG100 as well.

In order to re-sensitize antibiotic-resistant bacteria, EP inhibitors could be used to overcome the resistant phenotype. Ligand **3** was a potent EP inhibitor on MRSA but had no activity on the sensitive *S. aureus* reference strain and the Gram-negative *E. coli* AG100 expressing the AcrAB-TolC efflux system. The reason for that is the different cell wall composition, because Gram-positives have a thick layer of peptidoglycan, and in the case of MRSA isolate, the distribution of essential proteins involved in lipid metabolism, and the expression of EPs and membrane proteins may be different compared to sensitive ATCC isolates [21]. On the contrary, Gram-negatives possess a thin peptidoglycan layer, but because of the presence of the inner and outer membranes, tripartite efflux pump systems such as the AcrAB-TolC system can be overexpressed in resistant Gram-negative isolates. The Zn(II) complex **5** of ligand **2** and the Ag(II) complex **11** of ligand **1** inhibited the AcrAB-TolC efflux system in *E. coli* AG100, and these complexes exhibited moderate toxicity on normal MRC-5 human embryonal fibroblast cells (IC_50_ of 24.57 µM and 65.89 µM, respectively). According to previous studies [18], the Ag(II) complex **11** was found to exhibit the strongest inhibitory activity against various Gram-negative bacteria, especially against *Pseudomonas aeruginosa*, causing membrane permeabilization and degradation.

The bacterial virulence can be reduced if the bacterial communication and the virulence factors related to QS can be inhibited. The bacterial communication could be hampered by treatment with ligand **3**, the Ag(II) complex **11** of ligand **1**, and the Zn(II) complexes **4** and **5** of ligand **1** and **2**, respectively; however, the nature of the interaction between QS molecules and benzoxazole-based compounds needs further investigation. The QS-related process is the biofilm formation whereby microorganisms irreversibly attach to and grow on a surface and produce extracellular polymers that facilitate attachment and matrix formation, resulting in increased resistance towards antibiotic treatment. The most pronounced anti-biofilm activity was found in case of the Fe(III) complex **12** of ligand **1** on reference *S. aureus* and this complex had no toxicity on normal human fibroblast cells. Regarding antibacterial activity of the metal complexes, there were multiple modes of action. They could undergo ligand exchange reactions, release bioactive molecules, or be triggered by light irradiation to generate reactive oxygen species (ROS). However, complexes **4**–**12**, except the Ag(I) complex **11**, were fairly stable against air and moisture because the ligands **1**–**3** can possess a heteroaryl-substituted alkenol ligand system and act as the *N,O*-chelating ligand, producing highly stable metal complexes, which could be reinforced by a positive inductive effect of the heterocyclic moiety and a negative inductive effect of the CF_3_ group [18]. For example, Zn(II) complex **6** was inert to excess ethylenediaminetetraacetic acid (EDTA) at room temperature [18]. Therefore, these metal complexes may be stable in vivo. The increased potency on complexation of ligands with metals can be explained by the enhancement of the penetration into the lipid membranes due to the increased lipophilicity of the complexes [22,23].

Based on preliminary results, all compounds except Pd(II) complex **10** showed higher toxicity (IC_50_: 0.5–10.99 µM) against MDR mouse T-cell lymphoma cells than chemotherapeutic drug cisplatin (IC_50_: 11.20 µM) used as reference compound [19]. The present data demonstrated that Zn(II) complexes **4**, **5**, **6**, and Ni(II) complex **8** had a stronger inhibitory effect on MRC-5 human embryonic lung fibroblast cell line compared to the activity of cisplatin (IC_50_: 33.45 µM) [24] on the same cell line.

## 4. Materials and Methods

### 4.1. Compounds

The synthesis of three 2-trifluoroacetonylbenzoxazole ligands and their metal complexes (Zn, Cu, Ni, Mg, Pd, and Ag) was described previously [18] (Table 6). The Fe(III) complex **12** was obtained by the reaction of **1** with FeCl_3_ using the same method described for the preparation of **4** [18]. The compounds (**1**–**12**) screened for their antibacterial, MDR-modulating, anti-biofilm, and QS inhibition activities, furthermore, they were investigated in combination with antibiotics. The powdered compounds were dissolved in DMSO and stock solutions of 10 mM were prepared. The preparation was performed by vortexing at room temperature.

PMZ, CPZ, crystal violet (CV), EB, VER, TET, CIP, Luria–Bertani (LB) broth, and LB agar were obtained from Sigma–Aldrich Chemie GmbH (Steinheim, Germany). The modified LB medium (LB*) consisted of yeast extract 5 g/L, tryptone 10 g/L, NaCl 10 g/L, K_2_HPO_4_ 1 g/L, MgSO_4_ × 7H_2_O 0.3 g/L, and FeNaEDTA 36 mg/L. To obtain modified LB* agar, the LB* medium was enriched with agar 20 g/L (Difco, Detroit, MI, USA). The pH was adjusted to 7.2. Tryptic soy broth (TSB), tryptic soy agar, and Mueller Hinton broth (MHB) were obtained from Scharlau Chemie S.A. (Barcelona, Spain). CV solution was prepared by adding 0.05 g of CV to 50 mL of distilled water, then the solution was filtered (0.22 μM filter).

### 4.2. Bacterial Strains

Wild-type *E. coli* K-12 AG100 strain (argE3 thi-1 rpsL xyl mtl Δ(gal-uvrB) supE44) expressing the AcrAB TolC EP at its basal level and its AcrAB-TolC deleted mutant *E. coli* K-12 AG100 A strain. The strains were donated by Prof. Dr. Hiroshi Nikaido (Department of Molecular and Cell Biology and Chemistry, University of California, Berkeley, CA, USA).

The compounds were further investigated against two Gram-positive strains, *S. aureus* ATCC 25923 (MSSA), used as the methicillin susceptible reference strain, and the methicillin- and ofloxacin-resistant *S. aureus* 272123 clinical isolate (MRSA), which was given by Prof. Dr. Leonard Amaral (Institute of Hygiene and Tropical Medicine, Lisbon, Portugal).

The following bacterial strains were applied in the QS tests: *C. violaceum* 026 as sensor strain and *E. cloacae* 31298 isolated from clinical wound (used as N-acyl-homoserine lactone (AHL) producer); this strain induced pigment production by *C. violaceum* and is a model strain to study QS interactions.

### 4.3. Cell Culture

The MRC-5 human embryonic lung fibroblast cell line (ATCC CCL-171; provider: LGC Promochem, Teddington, UK) was used in the experiments. The cells were cultivated in Eagle’s Minimal Essential Medium (EMEM; Sigma–Aldrich, St. Louis, MO, USA), supplemented with 1% non-essential amino acid mixture, 10% heat-inactivated fetal bovine serum, 2 mM L-glutamine, 1 mM Na-pyruvate, nystatin, and a penicillin–streptomycin mixture in concentrations of 100 U/L and 10 mg/L, respectively. The supplementary components were obtained from Sigma–Aldrich, St. Louis, MO, USA. The cell culture was kept in a humidified atmosphere (5% CO_2_, 95% air) at 37 °C.

### 4.4. Determination of Minimum Inhibitory Concentrations

The minimum inhibitory concentrations (MICs) of compounds were determined in 96-well plates based on the Clinical and Laboratory Standard Institute guidelines (CLSI guidelines) [25]. The compounds were diluted in 100 μL of MHB. Then, 10^−4^ dilution of an overnight bacterial culture in 100 μL of medium were added to each well, with the exception of the medium control wells. The plates were further incubated at 37 °C for 18 h; at the end of the incubation period, the MIC values of tested compounds were determined by visual inspection.

### 4.5. Combination Effect

The chemosensitizing activity of benzoxazole-type ligands and their metal complexes was determined based on the MIC values of the antibiotics in the presence of sub-inhibitory concentrations of the compounds (¼ MIC) in both Gram-negative and Gram-positive strains. The MICs were determined in strains by the two-fold broth microdilution method in 96-well plates, using serial dilutions of TET and CIP. The first four rows contained two-fold dilutions of antibiotics, and combinations of the TET and CIP and tested compounds were transferred into the last four rows. The 10^−4^ dilution of an overnight bacterial culture in 50 μL of MHB was then measured to each well, except for the medium control wells. The plates were kept at 37 °C for 18 h. MIC values of antibiotics and their combination with tested compounds were detected by visual inspection.

### 4.6. Real-Time Accumulation Assay

The accumulation of the efflux pump substrate EB was monitored by the automated EB method [26] using a LightCycler real-time thermocycler (LightCycler 1.5, Roche, Indianapolis, IN, USA). An aliquot of an overnight culture of the *S. aureus* strains in TSB medium was inoculated into fresh TSB medium, and it was incubated until it reached an optical density (OD) of 0.6 at 600 nm. In the case of *E. coli* strains, the medium applied in the assay was LB broth; the preparation of the inoculum was similar to the one of *S. aureus*. The cells were washed with phosphate buffered saline (PBS; pH 7.4) and centrifuged at 13,000 × *g* for 3 min, the pellets were re-suspended in PBS (pH 7.4), and the OD was calibrated to 0.6 at 600 nm. The compounds were measured at ¼ MIC concentration (in double-concentrated form) to the EB solution in PBS. The final concentration of EB was determined according to the MIC and the fluorescent signal produced by this amount of EB. In the case of all strains, the concentration of EB was 1 μg/mL. Then, 10 μL of the EB solution plus the compound were pipetted into standard glass capillary tubes of 20 µL maximum volume (Roche, Indianapolis, USA), and 10 μL of bacterial culture (OD of 0.6 at 600 nm) were transferred to the capillaries. The capillaries containing the samples were placed into the carousel, and the fluorescence was monitored at the FL-2 channel in every minute on a real-time basis.

Based on the data obtained, the relative fluorescence index (RFI) of the last time point (minute 30) was determined as follows:

RFI = (RF_treated_ − RF_untreated_)/RF_untreated_ (1)

where RF_treated_ is the relative fluorescence (RF) at the last time point of the EB retention curve in the presence of an inhibitor, and RF_untreated_ is the relative fluorescence at the last time point of the EB retention curve of the untreated control having the solvent control (DMSO). VER was used as a positive control on MRSA strain and CPZ was used on Gram-negative and *S. aureus* ATCC strains.

### 4.7. Anti-Biofilm Effect

The biofilm production of the *S. aureus* ATCC 25923 strain was studied in 96-well plates using TSB broth in the presence of compounds. The overnight cultures were diluted to an OD of 0.1 at 600 nm and then measured to each well with the exception of the medium control wells, and compounds were pipetted individually at ¼ MIC concentration. The final volume was 200 μL in each well. The samples were incubated at 30 °C for 48 h with gentle agitation (100 rpm). Then, the medium was removed, and the plate was washed with tap water to discard unattached cells. 200 μL CV (0.1% [*v/v*]) was added to the wells and incubated for 15 min at room temperature. CV was removed from the wells and the plate was washed again with tap water. 200 μL 70% ethanol was transferred to each well and the biofilm formation was determined by measuring the OD at 600 nm using Multiscan EX ELISA reader (Thermo Labsystems, Cheshire, CT, USA). The anti-biofilm effect of compounds was determined as the percentage (%) of decrease in biofilm formation. The assay was repeated a minimum of three times. The results were calculated using a *t*-test and *p*-values of <0.001 were considered significant.

### 4.8. Assay for Quorum Sensing Inhibition

LB* was applied for these experiments. The sensor strain *C. violaceum* 026 and the AHL producer strains *E. cloacae* 31298 were inoculated as parallel lines and incubated at room temperature for 24–48 h. The inhibition of bacterial communication was determined by the agar diffusion method. Filter paper discs (7.0 mm in diameter) were impregnated with 10 μL of stock solutions (1 mM) of the compounds in DMSO. The discs were placed between the parallel lines of the sensor and the AHL producer strains. The plates were kept at room temperature for another 24–48 h, and the interactions between the strains and compounds were determined as the reduction in the size of the zone of purple pigment (violacein) production in millimeters [27]. PMZ was applied as a positive control.

### 4.9. Assay for Cytotoxic Effect

The activity of increasing concentrations of the compounds on cell growth was determined in 96-well flat-bottomed microtiter plates by two-fold serial dilutions of the benzoxazole derivatives. The adherent human embryonic lung fibroblast cells (10^4^/well) was seeded in EMEM medium in 96-well microtiter plates for 4 h prior to the assay. The serial dilutions of the compounds were made in a separate plate, and then pipetted to the plates containing the MRC-5 cell line. Culture plates were incubated at 37 °C for 24 h; at the end of the incubation period, 20 μL of MTT (3-(4,5-dimethylthiazol-2-yl)-2,5-diphenyltetrazolium bromide) solution (from a 5 mg/mL stock solution) was measured to each well. After the incubation at 37 °C for 4 h, 100 μL of sodium dodecyl sulfate (SDS) solution (10% SDS in 0.01 M HCl) was added to each well to dissolve the formazan crystals and the samples were further incubated at 37 °C overnight. Cell growth was calculated by measuring the OD at 540 nm (ref. 630 nm) with a Multiscan EX ELISA reader. The cytotoxicity was expressed as IC_50_ values, defined as the inhibitory dose that reduces the growth of the cells exposed to the tested compounds by 50%. IC_50_ values and the standard deviation (SD) of triplicate experiments were calculated by using GraphPad Prism software version 5.00 for Windows with nonlinear regression curve fit (GraphPad Software, San Diego, CA, USA; www.graphpad.com) where IC_50_: 0–30, IC_50_: 31–60, IC_50_: 61–100, and IC_50_ > 100 µM represent strong, moderate, weak, and no cytotoxic effects, respectively.

### 4.10. Statistical Analysis

The values were given as the mean ± SD determined for three replicates from three independent experiments. The analysis of data was performed using SigmaPlot for Windows Version 12.0 software (Systat Software Inc, San Jose, CA, USA), applying the two-tailed *t*-test.

## 5. Conclusions

It can be concluded that the 2-trifluoroacetonylbenzoxazole ligands and their metal complexes have various and potent antibacterial activities. Our findings are still preliminary because several issues should be analyzed and investigated in the future, e.g., in vivo stability of the complexes, their interaction with plasma proteins, toxicity on liver cells. However, these metal complexes could be potential and promising drug candidates applied alone or in combination with standard antibiotics in the treatment of infectious diseases caused by resistant bacteria.

## Figures and Tables

**Table 1:** Minimum inhibitory concentrations (MICs) of the ligands and their metal complexes on *Staphylococcus aureus* ATCC 25923, *S. aureus* 272123, *Escherichia coli* AG100, *E. coli* AG100 A, *Chromobacterium violaceum* 026, and *Enterobacter cloacae* 31298.

Compound	MIC (µM)					
	*S. aureus* ATCC 25923	*S. aureus* 272123 (MRSA)	*E. coli* AG100	*E. coli* AG100 A	*C. violaceum* 026	*E. cloacae* 31298
**1**	25	12.5	50	50	>100	>100
**2**	12.5	3.125	50	25	>100	>100
**3**	100	50	50	25	>100	>100
**4**	12.5	6.25	25	25	>100	>100
**5**	12.5	3.125	50	12.5	>100	>100
**6**	25	12.5	50	12.5	>100	>100
**7**	12.5	3.125	>100	12.5	>100	>100
**8**	25	6.25	50	25	>100	>100
**9**	25	6.25	50	25	>100	>100
**10**	>100	>100	>100	>100	>100	>100
**11**	25	12.5	25	50	>100	>100
**12**	50	25	>100	50	>100	>100

**Table 2:** Minimum inhibitory concentration (MIC) of ciprofloxacin (CIP) in combination with compounds in *Staphylococcus aureus* MRSA 272123 and *E. coli* AG100.

Compound ^1^	CIP_MIC_ (µM) Combination with Compounds	
	*S. aureus* MRSA	*E. coli* AG100
**3**	6.25	0.016
**5**	6.25	0.016
**7**	6.25	0.016
**8**	6.25	0.008
**9**	6.25	0.008
**12**	6.25	0.016
**CIP_MIC_**	12.5	0.016

^1^ The concentration of compounds was ¼ MIC.

**Table 3:** Relative fluorescence index (RFI) of tested compounds on *Staphylococcus aureus* ATCC 25923, *S. aureus* MRSA 272123, *E. coli* AG100, and *E. coli* AG100 A.

Compound	RFI			
	*S. aureus* ATCC 25923	*S. aureus* 272123 (MRSA)	*E. coli* AG100	*E. coli* AG100 A
**1**	−0.11	−0.14	−0.35	0
**2**	−0.07	−0.07	0.17	−0.01
**3**	−0.01	**0.13**	−0.07	−0.03
**4**	−0.03	−0.11	−0.10	−0.17
**5**	0.03	−0.13	**0.37**	−0.19
**6**	0.08	−0.02	0.08	−0.15
**7**	0.12	−0.01	−0.06	0.11
**8**	−0.20	−0.01	0.11	−0.15
**9**	−0.10	−0.01	−0.01	−0.03
**10**	0	0	−0.06	0
**11**	0.11	−0.11	**0.37**	0.12
**12**	−0.02	−0.02	0.04	0.09
**CPZ ^1^**	0.17	-	0.38	0.48
**VER ^2^**	-	0.14	-	-

^1^ Chlorpromazine; ^2^ verapamil. The active compounds are presented in bold.

**Table 4:** The quorum sensing inhibitory effect of the active compounds and the positive control promethazine (PMZ).

Compound	Inhibition Zone in mm	SD ^1^ (±)
**3**	56	3.3
**4**	47	2.1
**5**	16	0.9
**11**	53	1.4
**PMZ**	16	1.8

^1^ Standard deviation.

**Table 5:** Cytotoxic activity of tested compounds against MRC-5 human embryonal lung fibroblast cell line.

Compounds	MRC-5	
	IC_50_^1^ (µM)	SD ^2^ (±)
**1**	>100	-
**2**	>100	-
**3**	44.45	2.72
**4**	17.47	4.82
**5**	24.57	1.42
**6**	4.72	0.01
**7**	76.10	0.89
**8**	12.24	0.62
**9**	>100	-
**10**	89.11	6.02
**11**	65.89	3.8
**12**	>100	-

^1^ Inhibitory concentration 50; ^2^ standard deviation.

**Table 6:**
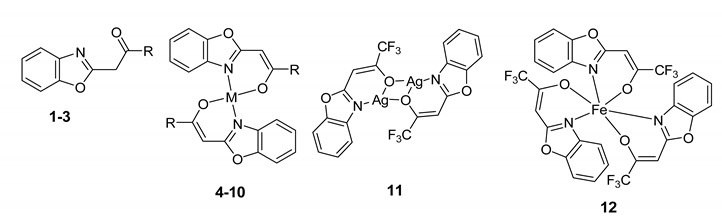
Structures of ligands and their metal complexes.

Compound	R	M	Structure ^1^	Molecular Weight
**1**	CF_3_	−	−	229
**2**	CF_2_Cl	−	−	245.6
**3**	CF_2_H	−	−	211
**4**	CF_3_	Zn	L_2_Zn	522
**5**	CF_2_Cl	Zn	L_2_Zn	554.6
**6**	CF_2_H	Zn	L_2_Zn	486
**7**	CF_3_	Cu	L_2_Cu	520
**8**	CF_3_	Ni	L_2_Ni	551 (apical 2H_2_O)
**9**	CF_3_	Mg	L_2_Mg	481
**10**	CF_3_	Pd	L_2_Pd	563
**11**	CF_3_	Ag	L_2_Ag_2_	672
**12**	CF_3_	Fe	L_3_Fe	740

^1^ L = ligand.

## References

[1] CDC (2019). Antibiotic Resistance Threats in the United States, 2019.

[2] Blair J.M.A., Webber M.A., Baylay A.J., Ogbolu D.O., Piddock L.J.V. (2015). Molecular mechanisms of antibiotic resistance. Nat. Rev. Microbiol..

[3] Blanco P., Hernando-Amado S., Reales-Calderon J.A., Corona F., Lira F., Alcalde-Rico M., Bernardini A., Sanchez M.B., Martinez J.L. (2016). Bacterial multidrug efflux pumps: Much more than antibiotic resistance determinants. Microorganisms.

[4] Reygaert W.C. (2018). An overview of the antimicrobial resistance mechanisms of bacteria. AIMS Microbiol..

[5] Wang-Kan X., Blair J.M.A., Chirullo B., Betts J., La Ragione R.M., Ivens A., Ricci V., Opperman T.J., Piddock L.J.V. (2017). Lack of AcrB efflux function confers loss of virulence on *Salmonella enterica* serovar Typhimurium. mBio.

[6] Santos A.L.S.D., Galdino A.C.M., de Mello T.P., de Ramos L.S., Branquinha M.H., Bolognese A.M., Columbano Neto J., Roudbary M. (2018). What are the advantages of living in a community? A microbial biofilm perspective!. Mem. Inst. Oswaldo Cruz.

[7] Abisado R.G., Benomar S., Klaus J.R., Dandekar A.A., Chandler J.R. (2018). Bacterial quorum sensing and microbial community interactions. mBio.

[8] Saxena P., Joshi Y., Rawat K., Bisht R. (2019). Biofilms: Architecture, resistance, quorum sensing and control mechanisms. Indian J. Microbiol..

[9] Patra M., Gasser G., Metzler-Nolte N. (2012). Small organometallic compounds as antibacterial agents. Dalton Trans..

[10] Barry N.P.E., Sadler P.J. (2013). Exploration of the medical periodic table: Towards new targets. Chem. Commun..

[11] Prachayasittikul V., Prachayasittikul V., Prachayasittikul S., Ruchirawat S. (2013). 8-Hydroxyquinolines: A review of their metal chelating properties and medicinal applications. Drug Des. Dev. Ther..

[12] Imramovsky A., Kozic J., Pesko M., Stolarikova J., Vinsova J., Kralova K., Jampilek J. (2014). Synthesis and antimycobacterial and photosynthesis-inhibiting evaluation of 2-[(*E*)-2-substituted-ethenyl]-1,3-benzoxazoles. Sci. World J..

[13] Zhang W., Liu J., Macho J.M., Jiang X., Xie D., Jiang F., Liu W., Fu L. (2017). Design, synthesis and antimicrobial evaluation of novel benzoxazole derivatives. Eur. J. Med. Chem..

[14] Wells G., Berry J.M., Bradshaw T.D., Burger A.M., Seaton A., Wang B., Westwell A.D., Stevens M.F.G. (2003). 4-substituted 4-hydroxycyclohexa-2,5-dien-1-ones with selective activities against colon and renal cancer cell lines. J. Med. Chem..

[15] Kawase M., Harada H., Saito S., Cui J., Tani S. (1999). In vitro susceptibility of *Helicobacter pylori* to trifluoromethyl ketones. Bioorg. Med. Chem. Lett..

[16] Kawase M., Motohashi N., Sakagami H., Kanamoto T., Nakashima H., Ferenczy L., Wolfard K., Miskolci C., Molnár J. (2001). Antimicrobial activity of trifluoromethyl ketones and their synergism with promethazine. Int. J. Antimicrob. Agents.

[17] Zoltan V., Armada A., Cerca P., Amaral L., Savka M.A., Szegedi E., Kawase M., Motohashi N., Molnár J. (2012). Inhibition of quorum sensing and efflux pump system by trifluoromethyl ketone proton pump inhibitors. In Vivo.

[18] Watanabe G., Sekiya H., Tamai E., Saijo R., Uno H., Mori S., Tanaka T., Maki J., Kawase M. (2018). Synthesis and antimicrobial activity of 2-trifluoroacetonylbenzoxazole ligands and their metal complexes. Chem. Pharm. Bull..

[19] Spengler G., Kincses A., Rácz B., Varga B., Watanabe G., Saijo R., Sekiya H., Tamai E., Maki J., Molnár J. (2018). Benzoxazole-based Zn(II) and Cu(II) complexes overcome multidrug-resistance in cancer. Anticancer Res..

[20] Frei A., Zuegg J., Elliott A.G., Baker M., Braese S., Brown C., Chen F., Dowson C.G., Dujardin G., Jung N. (2020). Metal complexes as a promising source for new antibiotics. Chem. Sci..

[21] Mouwakeh A., Kincses A., Nové M., Mosolygó T., Mohácsi-Farkas C., Kiskó G., Spengler G. (2019). *Nigella sativa* essential oil and its bioactive compounds as resistance modifiers against *Staphylococcus aureus*. Phytother. Res..

[22] Hunoor R.S., Patil B.R., Badiger D.S., Vadavi R.S., Gudasi K.B. (2010). 2D HETCOR studies of 1,2-dihydroquinazolinone derivative: Synthesis, characterization and anti-microbial study of its transition metal complexes. Pharm. Chem..

[23] Alaudeen M., Sushama P.G., Dorothy A.M. (2007). Synthesis and spectroscopic investigation of metal chelates of hydrazo pyrazolone derivative. Asian J. Chem..

[24] Gajdács M. (2018). Multidrug Resistance Reversing Activity of Organoselenium Compounds. Ph.D Thesis.

[25] Christopher P.J., Polgar E.P., CLSI (2015). Susceptibility Testing Process. Methods for Dilution Antimicrobial Susceptibility Tests for Bacteria that Grow Aerobically.

[26] Viveiros M., Martins A., Paixão L., Rodrigues L., Martins M., Couto I., Fähnrich E., Kern W.V., Amaral L. (2008). Demonstration of intrinsic efflux activity of *E. coli* K-12 AG100 by an automated ethidium bromide method. Int. J. Antimicrob. Agents.

[27] Varga Z.G., Szabó M.A., Schelz Z., Szegedi E., Amaral L., Molnar J. (2011). Quorum sensing inhibition by phenothiazines and related compounds. Lett. Drug Des. Discov..

